# Manual Delivery of the Plocenta

**Published:** 1849-04

**Authors:** 


### ECLECTIC DEPARTMENT,

### AND SPIRIT OF THE MEDICAL PERIODICAL PRESS.

*Manual Delivery of the Plocenta.*—By J. Byrd Smith, M. D., of Cincinnati.

The following is a portion of a communication in the Ohio Medical and Surgical Journal, No. for January, 1849:—

The method pursued by me, is during the expulsion of the fœtus, as the fundus becomes emptied, to make, or cause to be made, firm pressure over this part of the uterus. By this means, we insure a uniform and efficient contraction of the latter, from above, downwards, with the effect of detaching the placenta from its connection with the uterus during the same pains which expel the child. This kind of assistance, first suggested by Dr. Murphy, I have made use of in some 15 or 20 cases, and have never been disappointed in the result; the placenta, on examination, being found detached in all of them. After giving the necessary attention to the child, I proceed to an examination of the uterus, and if not found contracted, it is grasped firmly with both hands, and the compression continued until it is felt to harden and recede within the pelvis. Without waiting for pains, steady traction at the chord is pursued, whilst at the same time, the hand is grasping the uterus externally. Friction over the abdomen, I consider, for reasons before assigned, as not only inefficient, but sometimes, decidedly injurious. The operation of grasping the womb, is as readily performed, and much more likely to accomplish the desired object. After thus manipulating for a few minutes, if the placenta does not descend, I direct an assistant to make sufficient pressure externally, to maintain the fundus of the uterus, in a proper position, and proceed to pass the fingers, and if necessary, the hand, into the vagina, for the purpose of stimulating the uterus to contraction. Sometimes, this alone, excites contractions, but if not, the hand, being conically shaped, is passed along the funis, into the uterus; this should not be done in a hurry, but slowly and carefully, so as to avoid inflicting injury upon the woman. The hand being passed beyond the placenta, which will generally be found in the neck of the womb, grasps it. The increased volume of foreign matter in the cavity, is usually sufficient to produce immediate and efficient contractions, and the hand, with the secundines, will, in most instances, be forcibly expelled. If such is not the case, the hand containing the placenta, as much reduced in bulk by compression, as possible, is to be gently brought away; the assistant increasing the external pressure, as the womb is emptied of its contents. As soon as the placenta is removed, a bandage can be applied, and the woman made comfortable.

*Remarks, by Editor of Buffalo Medical Journal.*—The foregoing method of delivery of the placenta, we have practiced for the last ten years, or more, and had supposed it was peculiar to ourselves. Being at variance with the rules laid down by the best authorities in obstetrics, we have heretofore avoided any publication of our ideas on the subject; we have, however, been in the habit of expressing our opinion of the advantages of the practice in familiar intercourse with medical friends, but not, so far as we know, with the effect of its adoption by any of them. Finding, now, the same views enunciated in a communication by a respectable practitioner, we embrace the opportunity to state the results of our experience, which is much greater than that of the writer referred to, having extended over a period, of, at least, ten years.

The course which we invariably pursue, is as follows:—

Directly after the fœtus is expelled, or delivered, and the funis severed, we grasp, with one or both hands, the uterus above the pubis, and excite its contraction, by manipulation with the fingers, through the abdominal walls, varying the degree of pressure with the tenderness over the abdomen, never, of course, using force enough to occasion much, if any pain. The uterus will be readily felt contracting under the fingers, until, at length, it becomes firmly contracted. We then intermit our manipulations, and, if convenient, it is well for an assistant to make firm pressure over the lower part of the abdomen. Next, seizing the funis, we endeavor to follow up with the finger to its attachment to the placenta. Often this will be easy, the placenta being entirely detached, and lying in the os uteri, or in the vagina. We never make much, if any traction of the cord; but if the placenta be not readily felt, we proceed, with but little if any delay, gradually and carefully, to insinuate the fingers, arranged conically, into the vagina. In this procedure, we never give pain; if the patient complain in the least, we advance more slowly, or not at all. But in the vast majority of cases, the introduction of the fingers, and even the hand, (if it be not of a large size) is unattended by suffering. Generally, the fingers introduced will reach the placenta, or uterine contraction will be excited, which will bring the placenta within reach. If not, the hand is carried forward, provided no pain is occasioned. When the placenta is reached, we make careful efforts of extraction, hooking a finger over a portion of it, or plunging a finger into its structure. These efforts, and the presence of the hand, scarcely ever fail to excite contraction, and generally the necessity for continued manual delivery is slight—the accoucher has only to withdraw his fingers, or hand, before the advancing placenta, giving a little assistance if required.

The advantages of the delivery of the placenta in the manner just described, as it appears to us, are obvious. The patient is rendered comfortable, and in a condition to be put to bed, with less delay than when spontaneous expulsion is waited for. After the fœtus is expelled, there is always impatience and apprehension if the remainder of labor be protracted. The patient is fearful of something wrong, and will hardly be quieted by assurances to the contrary. The delay is vexatious to all parties. There is less danger of hemorrhage after the placenta has been removed, and the uterus has firmly contracted. Certainly, the manœuvre does not produce greater liability to hemorrhage. Since we have pursued the plan, we cannot recal a single case in which hemorrhage occurred to a degree to create anxiety. It is well known that when hemorrhage occurs, the placenta being retained, the most effectual method of arresting the flow of blood, is the manual delivery of the placenta. In those cases in which manual interference is required after waiting the length of time prescribed for the spontaneous conclusion of labor, the operation is rendered painful by the delay—the parts soon becoming sensitive and contracted. But directly after the delivery of the fœtus, while the parts are greaily relaxed, and the sensibility of the nerves obtunded, the manipulation is wholly unattended by suffering, provided proper gentleness be observed.

The only objection which seems to us to require notice, is the license which it may afford for roughness and force, by those who will look only to the end to be attained, without observing the requisite conditions. These conditions are, that no violence is ever to be used, and no efforts made, or continued, which occasion pain. But this is an objection which will apply to almost everything excellent. Abuses furnish no valid arguments against what is good or useful if properly used; or, to speak more properly, the danger of mal-practice should not obstruct the advantages of good practice.

